# Outcomes of Transcutaneous Retrobulbar Amphotericin B in Rhino-Orbital-Cerebral Mucormycosis Among Patients Recovering From COVID-19: A Preliminary Experience

**DOI:** 10.7759/cureus.27817

**Published:** 2022-08-09

**Authors:** Shivani Sinha, Vidya B Kumar, Abhay Kumar, Vivek Singh, Abhishek Anand, Rakhi Kusumesh, Sarita Mishra, Pragati Raj, Bibhuti P Sinha

**Affiliations:** 1 Ophthalmology, Indira Gandhi Institute of Medical Sciences, Patna, IND; 2 Medicine, Indira Gandhi Institute of Medical Sciences, Patna, IND; 3 Otolaryngology, Indira Gandhi Institute of Medical Sciences, Patna, IND

**Keywords:** post-covid-19 rhino-orbito-cerebral mucormycosis (rocm), ptosis, proptosis, exenteration, transcutaneous retrobulbar injection of amphotericin b (tramb)

## Abstract

Background

In this study, we aimed to assess the outcomes of transcutaneous retrobulbar injection of amphotericin B (TRAMB) in rhino-orbital-cerebral mucormycosis (ROCM) among patients recovering from coronavirus disease 2019 (COVID-19).

Methodology

This retrospective study was conducted at a tertiary care center in eastern India from May 29th to July 31st, 2021, and included post-COVID-19 patients admitted with stage 3 and 4a ROCM who underwent TRAMB. The details of the ophthalmic examination, laboratory investigations, and radiological examination were retrieved from patients records. Patients were given TRAMB (3.5 mg/mL) on alternate days till they underwent debulking surgery and resumed from the second postoperative day alternatively till the patients showed clinical stabilization or improvement.

Results

In total, 45 eyes of 41 patients were included in the study. The median number of injections given was six (minimum = 3; maximum = 10). Following was the distribution of number of injection needed in each eye: eight eyes (three injections), six eyes (four injections), seven eyes (five injections), three eyes (six injections), eight eyes (seven injections), 11 eyes (eight injections), and one eye had received nine and ten injections each. Overall, 21/32 (65.62%) eyes had improvement in proptosis whereas 9/32 (28.12%) had improvement in ptosis. Six patients had improvement in extraocular movement. In total, 25 eyes had no improvement whereas seven eyes had improvement in vision. Four eyes underwent exenteration. All nine patients with limited orbital disease had good improvement with fewer injections (median = 4). None of the patients undergoing TRAMB had an intracranial extension of disease. Moreover, 8.88% (4/45) of the eyes had post-TRAMB transient inflammation which resolved without any intervention. Finally, 3/41 of the patients died.

Conclusions

TRAMB can be considered as an useful therapeutic adjunct in managing ROCM. Further, it can halt the progression of the disease while awaiting definitive surgical intervention.

## Introduction

There has been a recent surge in the number of cases of rhino-orbital-cerebral mucormycosis (ROCM) during the second wave of the global coronavirus disease 2019 (COVID-19) pandemic. ROCM is a rapidly progressive and potentially lethal angioinvasive fungal sinusitis predisposed by immunosuppression. In Indians, the predisposing factors associated with mucormycosis include diabetes mellitus (DM), malignancy, and organ transplantation [1]. The increase in the number of mucormycosis cases in the Indian population appears to be due to the interaction of diabetes, indiscriminate use of corticosteroids, and COVID-19 [2].

The management of ROCM includes endoscopic sinus debridement, systemic antifungal therapy, and control of the underlying immunosuppression. Orbital involvement leads to intervention strategies such as orbital exenteration, conservative orbital debridement with or without irrigation with amphotericin B (AMB), and transcutaneous retrobulbar injection of amphotericin B (TRAMB) [3]. The fast-spreading nature of this fungus mandates urgent and aggressive intervention in suspected or confirmed cases of mucormycosis to achieve a satisfactory prognosis. Systemically administered antifungals have limited tissue penetrance due to the angioinvasive nature of the disease. Moreover, due to its high molecular weight, AMB diffuses slowly into tissues [4].

Local injection of TRAMB provides local administration to the infected tissues. Further, the use of retrobulbar injection of AMB has not been extensively studied in treating ROCM [5]. The use of TRAMB has been reported to have a lower risk of disfiguring exenteration without an apparent increase in the risk of mortality [6]. There is a lack of clarity regarding the indications and outcomes of TRAMB as a treatment modality as very few large-scale studies have been conducted among patients with ROCM recovering from COVID-19 [7]. This study was undertaken to assess the outcomes of TRAMB in ROCM among patients recovering from COVID-19.

## Materials and methods

This study adhered to the tenets of the Declaration of Helsinki and was approved by the Institutional Ethics Committee (231/IEC/IGIMS2021). This was a retrospective study undertaken at a dedicated tertiary care COVID-19 healthcare facility in eastern India. Informed written consent was obtained. The study was conducted from May 29th to July 31st, 2021. The patients were classified according to the staging proposed by Honavar et al. [8]. Patients recovering from COVID-19 admitted with stage 3 and 4a ROCM who underwent TRAMB were included in the study.

Patients with stage 3 ROCM were included in the study irrespective of the systemic comorbidities and best-corrected visual acuity. Patients with stage 4a ROCM were only included if the involvement of intraorbital structures was noted clinically and radiologically and not due to cavernous sinus thrombosis. Patients with disease restricted to paranasal sinuses (stage 1 and 2), stage 4b, 4c, and 4d ROCM were excluded from the study. Moreover, patients with no history of preceding COVID-19 illness were excluded from the study.

Demographic data and detailed history were obtained from patient records that included the history of COVID-19 infection, hospitalization, and medication. Patients had undergone complete general, otorhinolaryngological, ophthalmological examination, and radiological imaging. The following biochemical parameters were extracted: glycosylated hemoglobin, fasting blood sugar, complete blood count, C-reactive protein, and renal function test. Every patient had undergone nasal endoscopy. Nasal wash was sent for KOH and culture analyses to ascertain mucormycosis. Radiological imaging included contrast-enhanced magnetic resonance imaging (MRI), and the following findings were noted: involvement of intraocular muscles, intraorbital fat, orbital apex, and uptake of contrast. All patients included in the study had received intravenous liposomal AMB (5 mg/kg/day). The patients were planned for transnasal endoscopic orbital decompression or open surgical debridement.

Visual acuity was measured at the bedside using Snellen’s chart. The clinical evaluation of proptosis and ptosis was also done. Ocular motility was extracted for the horizontal and vertical gaze. The motility for each direction of gaze was evaluated on a scale from 0 (full excursion) to -4 (no excursion).

The patients were administered TRAMB (3.5 mg/ml) on alternate days until they underwent debulking surgery (endoscopic or open), and it was resumed from the second postoperative day alternatively until the patients showed clinical stabilization or improvement, whichever was earlier. The site of injection was guided by the MRI, toward the region of radiographic disease, usually along the medial orbital wall, whenever possible.

## Results

In total, 45 eyes of 41 patients were included in this study. The distribution of patients in different stages is depicted in Table 1. In total, 45 eyes underwent TRAMB on alternate days until patients underwent debulking surgery (endoscopic or open), and it was resumed from the second postoperative day alternatively until patients showed clinical stabilization or improvement, whichever was earlier. There were 17 females and 24 males, with a male preponderance of 58.5%. The mean and median age was 46 ± 2 years and 50 years (32-73 years), respectively. There was no difference in laterality between both eyes (p > 0.05%). The average duration between COVID-19 infection and the onset of ROCM was 20 ± 10 days.

**Table 1:** Distribution of patients among the different stages of ROCM. ROCM: rhino-orbital-cerebral mucormycosis

	Stage 3A ROCM	Stage 3B ROCM	Stage 3C ROCM	Stage 3D ROCM	Stage 4a ROCM
Number of patients	1	8	28	2	2
Number of eyes	1	8	28	4	4

The lymphocyte count was 9,284 ± 3,505 cells/mm^3^, neutrophil-to-lymphocyte ratio (NLR) was 5.97 ± 3.85, fasting blood sugar (FBS) was 301.86 ± 118.77 mg/dL, glycosylated hemoglobin was 10.24 ± 2.31%, and C-reactive protein was 47.23 ± 23.76 mg/dl. Overall, 90.24% (37/41) of the patients were diabetic, of whom 24 patients (64.86%) were recently diagnosed with DM post-COVID 19 infection. Six patients had co-existing comorbidities including kidney transplant and use of immunosuppressive therapy.

Only eight patients had a history of one dose of COVID-19 vaccination. Five vaccinated patients presented with stage 3A, two patients with stage 3C, and one patient with stage 4A ROCM.

The median number of injections given was six. The minimum and the maximum number of injections given were three and 10 respectively. The following was the distribution of the number of injections needed in each eye: eight eyes (three injections), six eyes (four injections), seven eyes (five injections), three eyes (six injections), eight eyes (seven injections), 11 eyes (eight injections), and one eye had received nine and ten injections each.

Overall, 36/45 (80.0%) of eyes had proptosis and ptosis at presentation. Four eyes underwent exenteration and were eliminated from the final assessment of the improvement of clinical parameters. Further, 21/32 (65.62%) eyes with proptosis improved clinically whereas 9/32 (28.12%) patients had improvement in ptosis. Proptosis and ptosis did not completely resolve during the study period. All eyes had restrictions of extraocular movement (EOM) in one or more gazes. Additionally, 6/41 (14.63%) patients had improvement in EOM but none of them recovered completely. Nearly 78.2% (32/41) of the patients had improvement in pain.

On presentation, 13/45 (28.88%) eyes had no perception of light (PL) due to ophthalmic artery or central retinal artery occlusion. In addition, 25 eyes had no improvement in vision. Seven eyes had improvement in vision. None of the PL-negative eyes had any improvement in vision. Different clinical parameters and their response to TRAMB have been outlined in Table 2.

**Table 2:** Different clinical parameters and their response to TRAMB. BCVA: best-corrected visual acuity; PL: perception of light; TRAMB: transcutaneous retrobulbar injection of amphotericin B

Parameters	Timelines and outcomes	Number of patients	Comments
Proptosis	At presentation	36/45	None of the parameters improved completely. Four eyes were excluded as they underwent exenteration
	Improvement	21/32	
Ptosis	At presentation	36/45	
	Improvement	9/32	
Extraocular movements	Restriction at presentation	45/45	
	Improvement	6/41	
BCVA	At presentation	PL-negative: 13/45	PL-negative eyes did not improve
	Improvement	7	

Four eyes underwent exenteration. Among them, two patients had stage 4a ROCM, and an attempt to salvage other eyes was made using TRAMB while two patients had stage 3c. Three eyes presented with ophthalmic artery occlusion with non-salvageable vision, and one patient with stage 3c had developed central retinal artery occlusion (CRAO) on postoperative day two of endoscopic debulking surgery. All nine patients with limited orbital disease (stage 3a and 3b) had a good improvement in all clinical parameters. They also needed fewer injections (median = 4).

None of the patients undergoing TRAMB injection had intracranial extension/worsening of disease during the course of the treatment. Overall, 8.88% (4/45) of the eyes had post-TRAMB transient inflammation which resolved without any intervention. At the end of the study period, 3/41 (7.31) of the patients died due to various systemic complications associated with pre-existing systemic comorbidities such as uncontrolled glycemic control and renal and cardiac failure.

## Discussion

The study was conceived for primarily two reasons. The first reason was to evaluate the outcomes of TRAMB in the management of ROCM among patients recovering from COVID-19. Additionally, we aimed to assess if it can be evaluated as a globe-sparing intervention, thereby lowering the morbidity of the disease. Due to the sudden surge in ROCM cases, there was a long waiting list of patients needing surgical debridement; hence, we needed more time for definitive surgical debridement.

Studies have reported a male preponderance ranging from 64.56-76% while we report a 58.5% male preponderance in our study [7,9-11]. The mean age of patients was 46 ± 2 years, and the median was 50 years (32-73 years), which is similar to that reported in the literature [7,11-15]. The mean duration from the diagnosis of COVID-19 to the diagnosis of mucormycosis was also comparable to other studies [11-13,15].

DM was reported in 93% of the patients with mucormycosis and COVID-19 in a systematic review [16]. Similarly, studies have reported DM in 74-100% of the patients with mucormycosis [2,9,17,18]. In our study, 90.24% (37/41) of the patients were diabetic, of whom 24 (64.86%) were recently diagnosed with diabetes post-COVID-19 infection. The FBS and mean glycosylated hemoglobin were suggestive of uncontrolled glycemic control. This highlights the importance of hyperglycemia and uncontrolled DM in the causation of mucormycosis in COVID-19. Patients with higher NLR ratio were found to have severe clinical stage and poor outcomes in mucormycosis [15].

Not many studies have reported the vaccination status of patients [19]. Eight (19.5%) patients had a history of one dose of vaccination, and the rest of the patients had no history of COVID-19 vaccination. The effect of vaccination on the course and prognosis of patients with ROCM recovering from COVID-19 remains unclear. Five (62.5%) vaccinated patients presented with stage 3A, two patients with stage 3C, and one patient with stage 4A ROCM. The majority of vaccinated patients (62.5%) presented with limited orbital disease, thereby favoring a good outcome with fewer injections. However, larger studies involving patients recovering from COVID-19 are needed to evaluate the effect of vaccination on the course and prognosis of ROCM.

Incorporating TRAMB for treating invasive fungal sinusitis with orbital involvement was reported to have a lower risk of disfiguring exenteration without an apparent increase in the risk of mortality [6]. There are limited data on the use of TRAMB in ROCM among patients recovering from COVID-19. Various case reports have highlighted the use of TRAMB in conjuction with systemic antifungals and endoscopic debridement [20,21]. A recent study reported TRAMB as an effective, economical, time-saving procedure in patients with orbital mucormycosis of mild-to-moderate severity [7].

There is a lack of large-scale studies involving TRAMB in the management of ROCM among patients recovering from COVID-19. A study in the Indian population incorporated TRAMB in the management of ROCM, in which the majority of eyes received three doses and a maximum of five injections [7]. In our study, the median number of injections given was six. The minimum and maximum number of injections given were three and ten, respectively. In our study, the following was the distribution of the number of injections needed in each eye: eight eyes (three injections), six eyes (four injections), seven eyes (five injections), three eyes (six injections), eight eyes (seven injections), 11 eyes (eight injections), and one eye received nine and ten injections each. Eyes that received nine and 10 injections had symptomatic relief in pain and improvement in clinical outcomes. Both the patients were stage 3c ROCM. All nine patients with limited orbital disease (stage 3a and 3b) had good improvement in all clinical parameters with fewer injections (median = 4).

In a previous study, only 6.09% of the patients [7] were reported to have improvement in proptosis compared to 65.62% of the patients who improved in our study. This can be attributed to the fact that every patient underwent orbital debridement (endoscopic/open). Ptosis and drooping of eyelids improved in 12.11-20.73% of the patients [7] compared to 28.12% of our patients. Both proptosis and ptosis did not completely resolve during the study period. One of the important clinical symptoms to get relieved was pain, and in two of our cases, the patient had repeated injections as they had significant reduction in pain.

All eyes had restriction of EOM. Six patients had improvement in EOM, which is similar to that reported earlier [7]. None of the patients with ophthalmoplegia recovered completely.

Overall, 62% of the eyes have been reported to be PL-negative in the literature [17]. In our study 26% of the patients presented with PL-negative vision, and 21.21% of the patients had improvement in vision. Visual improvement has been reported to be 3.57-7.31% in various studies [7,9]. It has been postulated that improved orbital disease control caused by retrobulbar treatment preserves perfusion to the optic nerve and leads to better visual outcomes [6]. None of the eyes recovered vision after presenting with no light perception.

A recent study has reported exenteration in approximately 5% of moderate ROCM cases to as high as 34.48% in severe cases [7]. Additionally, in a pre-COVID-19 era study, patients treated after incorporating TRAMB in the treatment algorithm were found to have lower risk of exenteration with similar mortality [6]. In a study by Nair et al., 80% of the globe could be salvaged in patients who received retrobulbar AMB injections [12]. Approximately 9.09% of the patients needed exenteration in our study which is lower than that reported in other studies. Four eyes of four patients underwent exenteration. Two of the patients had stage 4a ROCM while one patient had stage 3c and 3d each. Three eyes presented with ophthalmic artery occlusion and one patient with stage 3c developed CRAO on postoperative day two of endoscopic debulking surgery. In our study, a globe-sparing intervention was possible by incorporating TRAMB in our treatment algorithm in a majority of cases.

In view of the sudden surge in the number of ROCM patients, prompt orbital debridement was not possible for every case. A viable option was needed to slow down the progression of the disease while awaiting definitive surgical debridement. The results from our study are encouraging as TRAMB can be considered as an adjuvant therapeutic option in the management of ROCM.

None of the patients undergoing TRAMB injection had intracranial extension/worsening of disease during the course of the treatment. Overall, 8.88% of the eyes had post-TRAMB transient inflammation which resolved without any intervention but no serious ocular adverse effect was noted. The complication rate of TRAMB injection has been reported between 4.3% and 23.17% [6,7].

At the end of the study period, 3/41 (7.31%) of the patients died due to various systemic complications associated with preexisting systemic comorbidities such as renal failure, cardiac failure, and uncontrolled glycemic control unrelated to the status of ROCM.

The study limitations include the retrospective, nonrandomized design, the small sample size, and no control group. The patients included in the study also underwent sinus debridement and systemic AMB administration, which could have acted as confounding factors and led to a relatively good outcome.

## Conclusions

ROCM needs an urgent and timely intervention to decrease morbidity and mortality. TRAMB by administering AMB to the local site helps in controlling the disease. It can be considered a useful therapeutic adjunct in managing cases of ROCM as a globe-sparing intervention. Moreover, it can help halt the progression of the disease while awaiting definitive surgical intervention.
